# Minute ventilation sensor–driven rate response as a part of cardiac resynchronization therapy optimization in older patients

**DOI:** 10.1007/s10840-024-01848-1

**Published:** 2024-06-24

**Authors:** Jarkko Karvonen, Sanni Lehto, Corinna Lenz, Caroline Beaudoint, Sola Oyeniran, Torsten Kayser, Saila Vikman, Sami Pakarinen

**Affiliations:** 1 Department of Cardiology, Helsinki University Hospital Heart and Lung Center, Haartmaninkatu 4, 00029 Helsinki, Finland; 2 UKB Klinik Für Innere Medizin, Kardiologie, Berlin, Germany; 3 Boston Scientific, Green Square, Lambroekstraat 5D, 1831 Diegem, Belgium; 4 Heart Hospital, Tampere University Hospital, Tampere, Finland

**Keywords:** Cardiac resynchronization therapy, Minute ventilation, Chronotropic incompetence, Rate-response pacing, Heart rate score, Elderly patients

## Abstract

**Background:**

Chronotropic incompetence (CI) is common among elderly cardiac resynchronization therapy pacemaker (CRT-P) patients on optimal medical therapy. This study aimed to evaluate the impact of optimized rate-adaptive pacing utilizing the minute ventilation (MV) sensor on exercise tolerance.

**Methods:**

In a prospective, multicenter study, older patients (median age 76 years) with a guideline-based indication for CRT were evaluated following CRT-P implantation. If there was no documented CI, requiring clinically rate-responsive pacing, the device was programmed DDD at pre-discharge. At 1 month, a 6-min walk test (6MWT) was conducted. If the maximum heart rate was < 100 bpm or < 80% of the age-predicted maximum, the response was considered CI. Patients with CI were programmed with DDDR. At 3 months post-implant, the 6MWT was repeated in the correct respective programming mode. In addition, heart rate score (HRSc, defined as the percentage of all sensed and paced atrial events in the single tallest 10 bpm histogram bin) was assessed at 1 and 3 months.

**Results:**

CI was identified in 46/61 (75%) of patients without prior indication at enrollment. MV sensor–based DDDR mode increased heart rate in CI patients similarly to non-CI patients with intrinsically driven heart rates during 6MWT. Walking distance increased substantially with DDDR (349 ± 132 m vs. 376 ± 128 m at 1 and 3 months, respectively, *p* < 0.05). Furthermore, DDDR reduced HRSc by 14% (absolute reduction, *p* < 0.001) in those with more severe CI, i.e., HRSc ≥ 70%.

**Conclusion:**

Exercise tolerance in older CRT-P patients can be further improved by the utilization of an MV sensor.

**Graphical Abstract:**

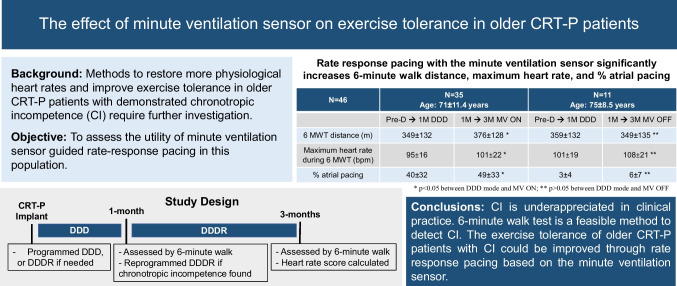

**Supplementary Information:**

The online version contains supplementary material available at 10.1007/s10840-024-01848-1.

## Introduction

The prevalence of advanced heart failure (HF) is progressively increasing worldwide. It is estimated that the prevalence of HF will increase by 46% between 2010 and 2030, probably related to longer life expectancy [1]. Cardiac resynchronization therapy (CRT) is a valid therapeutic option for patients with systolic HF, long QRS duration (QRSD), and optimal medical therapy [2]. CRT in elderly patients has also become increasingly common in clinical practice. Even though geographical differences may exist, CRT-P tends to be more commonly used in elderly patients than CRT-D. In a nationwide HF registry, 72.6% of CRT-P recipients were ≥ 70 years of age, whereas only 43.2% of CRT-D patients were in this age group [3]. Older patients with more comorbidity, cognitive dysfunction, or frailty are underrepresented in clinical trials that assess the effectiveness of CRT. Thus, the elderly population is not well represented in the guidelines [4]. However, patients older than 75 years have similar benefits from CRT as patients younger than 75 years, with equivalent CRT response rates [5, 6].

With greater optimized pharmacological treatment of HF, especially the use of beta-blockers, more patients are affected by chronotropic incompetence (CI). Furthermore, the autonomic nervous system is chronically shifted toward the sympathetic pathway in patients with HF and has been shown to reduce β-adrenergic responsiveness, resulting in a reduced heart rate (HR) response during exercise in spite of the typically elevated resting HR [7]. CI is generally defined as the inability to increase HR adequately during exercise to match cardiac output to metabolic demands. CI in HF is associated with reduced functional capacity [8] and poor survival [9]. In a healthy heart, HR, stroke volume, and cardiac output increase during exercise, whereas in a failing heart, contractility reserve is lost, thus rendering increases in cardiac output primarily dependent on the increase in HR. Consequently, insufficient increases in HR because of CI may be considered a major limiting factor in the exercise capacity of patients with HF [10].

Rate-adaptive pacing as a pacemaker feature has been used in clinical routine for patients with CI to restore physiological HR response to daily physical activities. Assuming a causal link between CI and the limitation in exercise capacity in patients with HF, the reversal of CI by rate-adaptive pacing should increase exercise capacity. Thus, CI serves as a possible therapeutic target using implantable cardiac device technology in patients with HF. Despite the potential importance of CI in HF, the issue has drawn limited attention and is often unrecognized in clinical practice. Rate-adaptive pacemakers control HR using a single activity sensor or a combination of sensors. Studies investigating the effects of different types of rate-adaptive pacing modes in different stages of CI have shown variable results, thus further studies are needed. Measuring respiratory minute ventilation (MV) offers a physiological approach to assessing metabolic activity, including high specificity, good proportionality to metabolic needs, and high sensor reliability but with a moderate speed of response [10]. To our knowledge, there is no previous data available on the use of the MV sensor alone in HF patients with CRT.

**Fig. 1:**
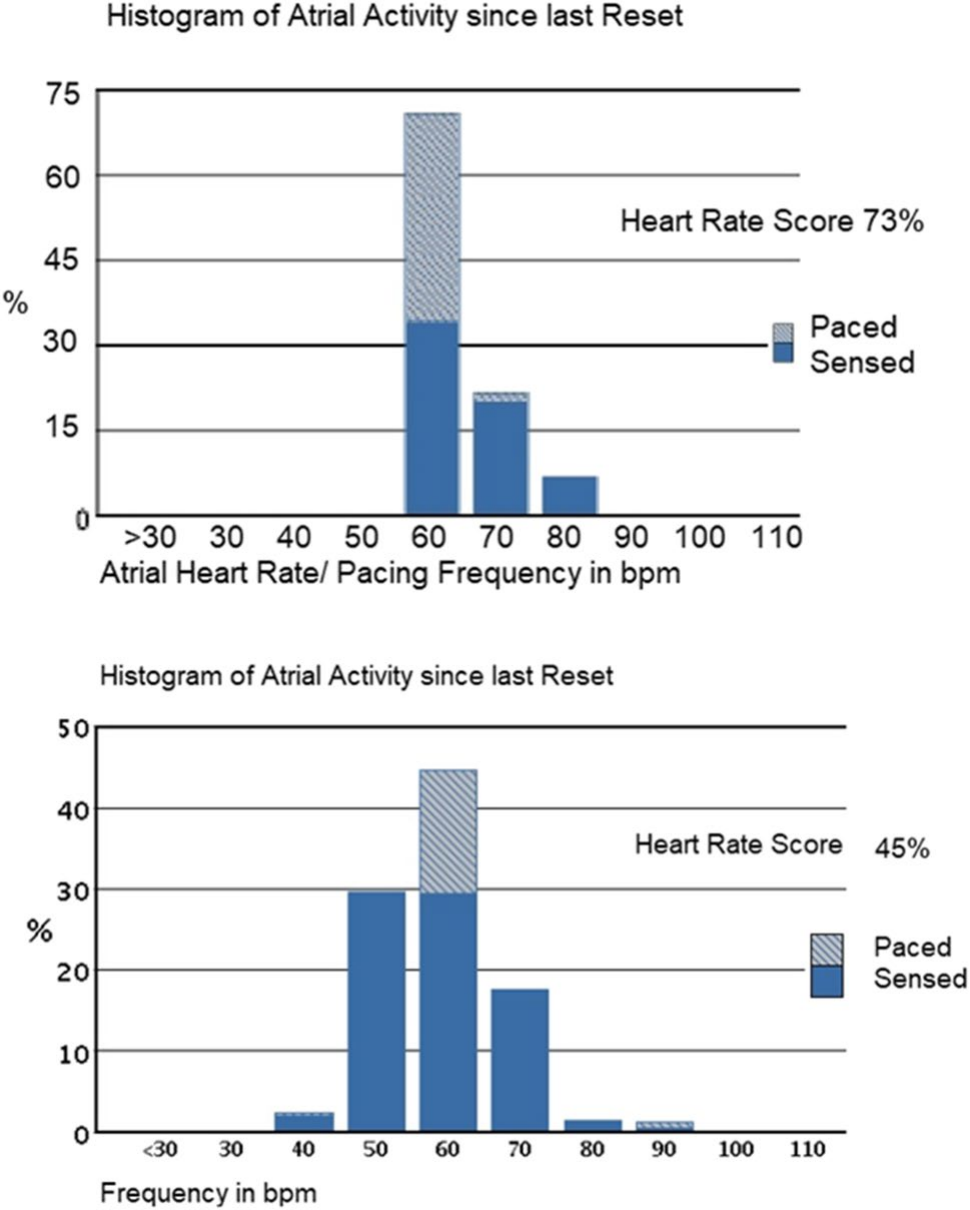
Two examples of the heart rate score (HRSc) calculation based on in-clinic or remotely available diagnostic device data. The score corresponds to the percentage of atrial activity in the largest 10 bpm histogram bin

A substantial part of the HF population with reduced left ventricular ejection fraction (LVEF) is currently implanted with a cardiac implantable electronic device, which offers a unique opportunity to study HR dynamics. An option for identifying CI during in-clinic or remote follow-up is the use of a HR Score (HRSc), which allows using a common heart rate histogram as a marker of chronotropic performance [11]. The HRSc is defined as the percentage of all sensed and paced atrial events in the single tallest 10 bpm histogram bin (Fig. 1). For example, when all events occur in the 60 to 70 bpm bin, the HRSc is 100%. When events are distributed over a wider range with rates < 60 bpm and > 70 bpm, the HRSc becomes lower. Using a cutoff value of 70%, it has been demonstrated that a HRSc ≥ 70% independently predicts 5-year mortality in a large population of CRT-D patients [12]. This is one of several variables within our awareness correlating with survival. Other factors include for example indication at enrolment (LBBB preferable for CRT) [13], percentage of biventricular pacing [14], and adherence to guideline-directed medications [15].

In CRT patients with CI and a HRSc ≥ 70%, reprogramming the device from dual chamber (DDD) mode to dual chamber rate-adaptive (DDDR) mode (i.e., rate-adaptive pacing ON) improved (i.e., lowered) the HRSc and was associated with improved survival. Rate-adaptive pacing has thus shown favorable effects on both exercise capacity and survival in a well-selected subset of HF patients with CI, although the retrospective nature of the study limits the interpretation of the results [12]. Advances in device technology by incorporating additional physiological activity sensors, like the MV sensor, and the detection of CI using a device histogram-based score, such as the HRSc, might improve future treatment of CI in the HF population.

Finally, two indicators have been associated with better CRT response in clinical trials: prolonged interventricular delay, as measured by the difference in activation time (V-V timing) between the right ventricular (RV) sensing electrode and left ventricular (LV) sensing electrode [16–18], and shortening of the QRS duration (∆QRSD) following CRT system implantation [19, 20]. The main objective of our study was to assess the impact of optimized rate-adaptive pacing on exercise tolerance with CRT-P devices in an elderly population using the MV sensor alone in a prospective clinical trial setting. The secondary objective was to determine if DDDR mode with the use of an MV sensor (DDDR-MV) improves HRSc in elderly CRT-P patients. We also analyzed the correlations between exercise tolerance, HRSc, V-V timing, and ∆QRSD.

## Methods

### Study design

The Rally CRT-P study (NCT02488239) was a prospective, multicenter trial of patients with a well-established CRT-P indication, following the ESC guidelines. Patients with symptomatic HF (NYHA class II or III) and a successfully implanted VISIONIST CRT-P device (de novo or upgrade) were assessed.

The main inclusion criteria were as follows:

- Planned to be implanted or replaced with a VISIONIST Ingenio 2 CRT-P device
- Planned to be implanted with a 3-lead CRT-P system
- Planned to be connected to the remote data collection through the LATITUDE® system
- Able to do a 6-min walk test (6MWT)
- Maximum sensor rate of age-predicted maximal heart rate (APMHR) 80% should be clinically acceptable

The device system was equipped with a rate-response sensor to increase HR based on MV. The programming allowed the device to measure the electrical delay between RV and LV sensing electrodes. Data was collected on demographics, resting and maximum HR, percentage of atrial pacing, sensing delay between RV and LV electrodes (V-V delay), device programming, walking distance, and adverse events. All data collection requirements including device-related measurements are shown in Supplementary Table 1. Patients with a known need for rate-response pacing based on medical history were maintained with rate-response ON at pre-discharge. At 1 month, patients were assessed by 6MWT, including those with and without known CI, with CI defined as HR trend < 100 bpm or 6MWT peak HR < 80% APMHR [21–25]. Patients identified with CI underwent a programming change from DDD to DDDR; patients not meeting the criteria for CI remained DDD. At 3 months post-implant, an additional 6MWT was performed.

Sensor optimization was individually done per patient at 1-month follow-up and followed the protocol below:

Prior to the initial device interrogation, subjects did a 6-min brisk walk, in a non-rate-adaptive pacing mode.

The interrogation of subjects’ devices occurred following the completion of the 6MWT. Patients were classified as CI subjects based on rate trend diagnostics for the previous 24 h (including the 6MWT). If the maximum heart rate was < 100 bpm or < 80% of the age-predicted heart rate ([220 − age] × 80%), the MV sensor was programmed ON (pacing mode DDDR if the patient was in sinus rhythm). The accelerometer was turned off during the course of the study until the 12th month of LATITUDE close-out follow-up. In case the accelerometer needed to be turned on for clinical reasons, an event and corrective action had to be documented in the study database. Rate-adaptive pacing during this study should be triggered by the MV sensor only.

Optimization guidance is as follows:

- The maximum sensor rate should be programmed to APMHR × 80%.
- The maximum sensor rate should not be programmed below 110 bpm.
- The MV “Response Factor” should be programmed based on the result of the sensor modulation after 6MWT, starting at a nominal value of 8.
- The resulting HR frequency in the sensor response modulation (especially in the 2nd part of the 6MWT) should result in a minimum of 70% of APMHR so that an appropriate HR can be achieved during future exercises.

### Heart rate score analysis

**Fig. 2:**
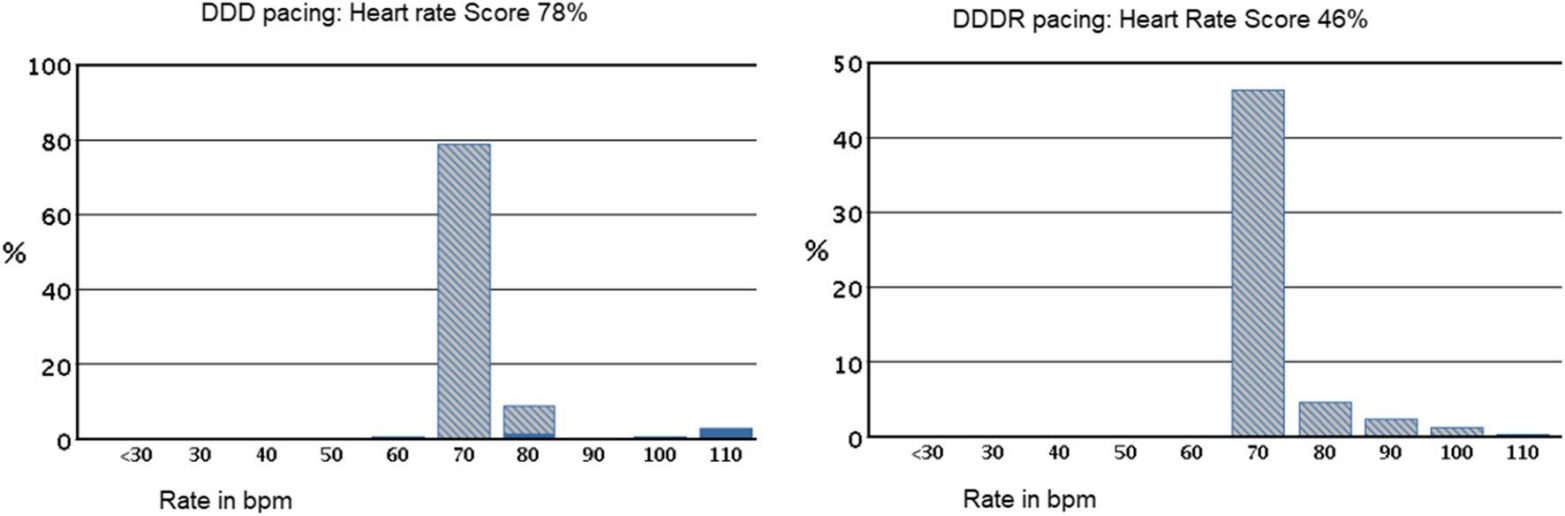
An example of patient heart rate score with device programmed DDD (left) and following programming to DDDR (right)

HRSc was measured for patients who were programmed to DDD between pre-discharge and 1-month follow-up and were found to have CI at follow-up (Fig. 2). From 1 to 3 months of follow-up, those patients were programmed to DDDR and remained mainly in sinus rhythm. At 3 months post-implant, HRSc was collected from the device, and the programming impact on HRSc between 1 and 3 months of follow-up was determined. Patients were dichotomized at a HRSc of 70%, and the result of the 6MWT between patients with HRSc > / < 70% was compared.

### Implantation and measurements

Devices and leads used in the study were fully commercially available, and all patients were planned to receive a CRT-P implant as part of their standard of care. The assignment of the specific Ingenio 2 CRT-P VISIONIST device was the physician’s choice as well as the consideration to use leads currently in place from previous devices and/or to use planned new leads (e.g., ACUITY X4 and/or other LV leads). Given the well-established clinical field experience with this pacing platform, no additional risks were expected when compared to implantation and follow-up procedures associated with any commercially available CRT-P device. The difference between biventricular paced and preimplantation QRS width was calculated. Electrocardiogram (ECG) measurements were made using any lead to obtain the largest value. The QRSD was defined as the interval between the earliest onset of the QRS waveform in any ECG lead and the latest offset in any lead. In the case of paced beats, pacing spikes were not considered the onset of the QRS complex. At 1 and 3 months, 6MWT was assessed in patients with and without known CI.

### Statistical analyses

Continuous variables are expressed as means ± standard deviation. Between-group comparisons were made by Mann–Whitney’s *U*-test for continuous variables and by Fisher’s exact test for contingency. Correlations were measured with Spearman’s rank correlation coefficient. A *p*-value < 0.05 was considered statistically significant.

## Results

### Patient characteristics and disposition

**Fig. 3:**
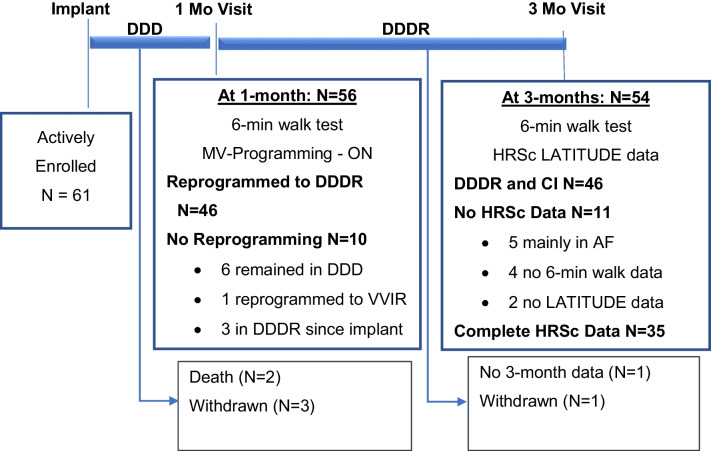
Patient follow-up overview during the Rally CRT-P study and selection of patients with sufficient data for CI identification and high heart rate score based on atrial pacing and sensing

Of 64 enrolled subjects, 61 were implanted with a CRT-P. Fifty-seven devices were programmed DDD, three devices were programmed DDDR, and one device was set to VVIR at implant. The disposition of patients throughout the study is shown in Fig. 3. Seventeen patients had a prior pacemaker implant and received an upgrade to CRT-P therapy. One patient received a CRT-P replacement device. Sixty patients received a quadripolar lead. The demographics of the study patients are shown in Table 1. The median age of the study population was 76 years with a mean LVEF of 41.1 ± 11.6% and an NYHA class score of 2.6 ± 0.64. There were no differences in sex, height, weight, or BMI. Out of 61 enrolled patients successfully implanted, 56 presented at 1-month follow-up and were tested for CI with a 6MWT. Forty-six were determined to have CI and consequently reprogrammed to DDDR mode (using MV sensor only). Eleven patients were classified as non-CI and programmed to DDD after 6MWT. A subset of six non-CI patients remained in DDD pacing mode and had LATITUDE data available, 3 patients were previously programmed DDDR at pre-discharge, and 1 patient was in persistent atrial fibrillation (AF) and programmed to VVIR. Fifty-four patients completed follow-up at 3 months, including the 46 patients who needed additional DDDR programming at 1 month.

**Table 1:** Selected patient characteristics and clinical history

Demographics and clinical history	*N*	*N* (%) or median**
Age at informed consent (year)**	61	76
Height (cm)**		170
Weight (kg)**		80
BMI (kg/m^2^)**		28
Resting heart rate (bpm)**		64
Sex female		24 (39%)
NYHA class	60	
Class I		3 (5%)
Class II		19 (32%)
Class III		36 (60%)
Class IV		2 (3%)
History of any AV-block*	61	35 (57%)
History of paroxysmal AF*		23 (38%)
Hypertension*		40 (66%)
Diabetes mellitus*		22 (36%)
Renal disease*		20 (33%)
Cardiac rhythm at visit prior to implant		
Normal sinus rhythm		40 (66%)
Paced		12 (20%)
Other		4 (7%)
Atrial fibrillation		4 (7%)
QRS morphology	59	
Normal		18 (31%)
Right bundle branch block		5 (8%)
Left bundle branch block		34 (58%)
Other conduction disorder		2 (3%)
Intrinsic QRS width (ms)**	57	150
Intrinsic PR interval (ms)**	46	195
Intrinsic QT interval (ms)**	54	457
LVEF (%)**	59	40
Ischemic cardiomyopathy	61	34 (56%)

Percentages are based on available observations (*N*)

*AF* atrial fibrillation, *BMI* body mass index, *bpm* beats per minute, *LVEF* left ventricular ejection fraction, *NYHA* New York Heart Association

^*^Diseases were not mutually exclusive. Percentages are based on the available observations (*N*). **Median values are given for all continuous variables

### HRSc analysis

In total, 46 subjects with DDDR programming and CI were identified as candidates for later remote analysis of HRSc.

HRSc and 6MWT were successfully analyzed in 35/61 patients (57%) (Fig. 3), following the exclusion of 5 patients mainly in AF for whom HRSc could not be calculated, 4 patients who did not complete the 6MWT, and 2 patients with no LATITUDE data available. Patients with CI and the need for sensor-supported adjustment of pacing frequency were only identified due to additional testing at 1-month follow-up.

The impact of sensor-supported programming on HRSc and walking distance in this cohort was studied based on the comparison of 1-month and 3-month data. More severe CI with HRSc ≥ 70% was present in 14/61 (23%) patients, and 21/61 were CI with HRSc < 70% (Table 2). At 1-month follow-up, 35 patients had MV sensor turned ON, 11 patients remained in non-rate-adaptive pacing mode (DDD), and 15 patients were excluded (no complete dataset due to early study withdrawal or missed follow-up visits or patients with other programming) (Fig. 3).

**Table 2:** Patient baseline data for all CI patients (*N* = 35) in sinus rhythm and with complete data sets at 3-month follow-up, comparing patients with HRSc ≥ 70% versus < 70%

Baseline characteristics	Heart rate score in all CI patients			*p*-value
	Measurement	< 70%	≥ 70%	
Age (Years)	(*N*) median	(21) 71	(14) 76	0.043*
LVEF (%)	(*N*) median	(21) 45	(14) 38	0.49
Height (cm)	(*N*) median	(21) 170	(14) 170	0.94
Weight (kg)	(*N*) median	(21) 79	(14) 80	0.84
BMI (kg/m^2^)	(*N*) median	(21) 27	(14) 28	0.91
Systolic blood pressure (mmHg)	(*N*) median	(21) 128	(14) 145	0.009*
Diastolic blood pressure (mmHg)	(*N*) median	(21) 76	(14) 80	0.31
Sex, *N* (%)	Female	9/21 (43%)	5/14 (36%)	0.67
Etiology, *N* (%)	Ischemic Cardiomyopathy	12/20 (60%)	8/14 (57%)	0.87
NYHA class, *N* (%)	II	8/20 (40%)	4/14 (29%)	0.28
	III	11/20 (55%)	8/14 (57%)	
	IV	N/A	2/14 (14%)	
QRS morphology, *N* (%)	LBBB	10/21 (48%)	9/14 (64%)	0.23
	Normal	9/21 (43%)	2/14 (14%)	
	Other	N/A	1/14 (7%)	
	RBBB	2/21 (9%)	2/14 (14%)	
Beta-blocker, *N* (%)	Yes	16/21 (76%)	10/14 (71%)	0.75
Intrinsic QRS width (ms)	(*N*) median	(20) 141	(13) 156	0.12
Intrinsic PR interval (ms)	(*N*) median	(19) 196	(13) 196	0.67

Percentages are based on available observations (*N*)

*BMI* body mass index, *LBBB* left bundle branch block, *LVEF* left ventricular ejection fraction, *NYHA* New York Heart Association, *RBBB* right bundle branch block

^*^Statistically significant

Table 3 shows clinical history data at baseline (prior device implant) for patients programmed to DDD and DDDR after 6MWT at 1-month follow-up determining the need for sensor programming according to protocol guidance.

**Table 3:** Clinical data at baseline obtained prior to CRT-P implantation. The 6-min walk test at 1-month follow-up determined MV sensor programming (ON/OFF) until 3-month follow-up. Patients with < 80% of age-predicted HR during 6-min walk test were programmed to DDDR

Baseline characteristic at enrollment	Measurement	Non-CI (DDD) patients at 1 month (*N* = 11)	CI (DDDR-MV) patients at 1 month (*N* = 35)	*p*-value
Age at informed consent (year)	Median (range)	79 (60–86)	75 (46–86)	0.28
BMI (kg/m^2^)	Median (range)	28.4 (23.7–36.1)	27.0 (21.8–46.9)	0.62
Sex [*N* (%)]	Female	3/11 (27.3%)	13/35 (37.1%)	0.55
NYHA class [*N* (%)]	I–II	4/11 (36.4%)	15/35 (42.9%)	0.56
	III–IV	7/11 (63.6%)	19/35 (54.3%)	
Resting heart rate	Mean ± range	71.5 ± 15.6	65.4 ± 16.0	0.27
LVEF in %	Mean ± range	44.3 ± 15.4	39.8 ± 10.4	0.27
Ischemic cardiomyopathy [*N* (%)]	Yes	7/11 (63.6%)	20/35 (57.1%)	0.78
Systolic blood pressure (mmHg)	Mean ± range	135.6 ± 22.8	137.4 ± 19.3	0.80
Ischemic cardiomyopathy [*N* (%)]	Yes	7/11 (63.6%)	20/35 (57.1%)	0.78
Is the subject on stable drug treatment? [*N* (%)]	Yes	11/11 (100.0%)	34/35 (97.1%)	0.57
Angiotensin-converting-enzyme (ACE) inhibitor [*N* (%)]	Yes	3/11 (27.3%)	23/35 (65.7%)	0.025*
Angiotensin II (ATII) receptor antagonist [*N* (%)]	Yes	4/11 (36.4%)	7/35 (20.0%)	0.27
Antiarrhythmic [*N* (%)]	Yes	1/11 (9.1%)	3/35 (8.6%)	0.96
Anticoagulant [*N* (%)]	Yes	5/11 (45.5%)	18/35 (51.4%)	0.73
Antiplatelet [*N* (%)]	Yes	3/11 (27.3%)	13/35 (37.1%)	0.55
Aldosterone antagonist [*N* (%)]	Yes	1/11 (9.1%)	5/35 (14.3%)	0.66
Beta-blocker [*N* (%)]	Yes	4/11 (36.4%)	25/35 (71.4%)	0.036*
Digitalis [*N* (%)]	Yes	0/11 (0.0%)	0/35 (0.0%)	NA
Diuretics [*N* (%)]	Yes	10/11 (90.9%)	21/35 (60.0%)	0.056
Statins [*N* (%)]	Yes	6/11 (54.5%)	25/35 (71.4%)	0.30
Calcium antagonists [*N* (%)]	Yes	3/11 (27.3%)	3/35 (8.6%)	0.11
In chronic AF [*N* (%)]	Yes	0/11 (0.0%)	0/35 (0.0%)	
AV-block [*N* (%)]	Yes	7/11 (63.6%)	23/35 (65.7%)	0.56
V-V time (ms)	*N*	5	20	0.37
	Median (range)	85.0 (8.0–95.0)	97.5 (13.0–148.0)	
Intrinsic QRS width (ms)	*N*	11	33	0.39
	Median (range)	142 (66–188)	156 (70–250)	
Intrinsic PR interval (ms)	*N*	7	31	0.78
	Median (range)	210 (116–314)	194 (100–464)	
Intrinsic QT interval (ms)	*N*	11	31	0.87
	Median (range)	454 (350–536)	460 (300–602)	

*BMI* body mass index, *LVEF* left ventricular ejection fraction, *NYHA* New York Heart Association

^*^Statistically significant

No MV sensor–related adverse events were reported. With DDDR-MV programming, there was a substantial increase in percent atrial pacing, maximum HR, and walking distance. Atrial pacing demonstrated a comparable increase in maximum HR among CI patients when compared to non-CI patients with intrinsic atrial responses (Table 4). In CI patients, DDDR-MV resulted in a 6% absolute reduction in HRSc (*p* = 0.02). In patients with documented CI during 6MWT and a HRSc ≥ 70% (based on device diagnostics), this reduction was even more pronounced at 14% (*p* < 0.001). HRSc was not reduced in non-CI patients (*p* = 0.29) or if HRSc was < 70% (*p* = 0.68). Waking distance increased, but not substantially, with DDDR-MV irrespective of the HRSc (Table 5).

**Table 4:** Impact of MV-driven atrial pacing on resting heart rate, maximal heart rate, 6-min walk distance, and percentage of atrial pacing in chronotropic incompetent patients with “MV ON” and “MV OFF” between 1- and 3-month follow-ups in comparison to baseline

*N* = 46	*N* = 35		*N* = 11	
	Age: 71 ± 11.4 years		Age: 75 ± 8.5 years	
	Pre-D → 1 M	1 M → 3 M	Pre-D → 1 M	1 M → 3 M
	DDD	MV ON	DDD	MV OFF
APMHR 80% (bpm)	120 ± 8		116 ± 7	
Resting heart rate (bpm)	72 ± 11	70 ± 10	74 ± 6	70 ± 9
Maximum heart rate during 6 MWT (bpm)	95 ± 16	101 ± 22*; + 6%; ↗ in 20/35 pts	101 ± 19	108 ± 21**; ↗ in 7/11 pts
6 MWT distance (meters)	349 ± 132	376 ± 128*; increased + 8%	359 ± 132	349 ± 135**
% Time with atrial pacing	40 ± 32	49 ± 33*; increased + 22%	3 ± 4	6 ± 7**

*6MWT* 6-min walk test, *APMHR* age-predicted maximal heart rate, *bpm* beats per minute, *MV* minute ventilation, *Pre-D* pre-discharge

^*^p < 0.05 between DDD mode and MV ON; **p > 0.05 between DDD mode and MV OFF

**Table 5:** Impact of HRSc on patient 6-m walk test heart rate and walking distance for all CI patients mainly in sinus rhythm and meeting per protocol data collection (*N* = 35)

Measure	Follow-up visit	HRSc data MV ON at 1 month	HRSc data sets changed at 1 month to MV ON		Comparing HRSc cohorts
			HRSc < 70%	HRSc ≥ 70%	*p*-value
*N*		35	21	14	
80% APMHR (bpm)		119 ± 7	122 ± 8	116 ± 5	0.033*
6-min walk heart rate (bpm)	1 month	96 ± 16	100 ± 16	89 ± 15	0.035*
	3 months	103 ± 23	110 ± 21	94 ± 22	0.044*
*p*-value on delta	Delta	0.017	0.036	0.28	
6-min walk distance (meters)	1 month	342 ± 119	340 ± 127	344 ± 110	0.92
	3 months	380 ± 135	385 ± 149	372 ± 115	0.79
*p*-value on delta	Delta	0.006	0.031	0.095	
Heart rate score (%)	1 month	64 ± 20	50 ± 12	84 ± 8	< 0.001*
	3 months	59 ± 17	51 ± 14	70 ± 14	< 0.001*
*p*-value on delta	Delta	0.023	0.68	< 0.001	
Heart rate prior to walk test (bpm)	1 month	71 ± 11	75 ± 11	66 ± 7	
	3 months	70 ± 10	72 ± 9	65 ± 9	
Atrial pacing (%)	1 month	38 ± 32	17 ± 16	67 ± 23	
	3 months	47 ± 33	31 ± 29	73 ± 18	

*APMHR* age-predicted maximal heart rate, *bpm* beats per minute, *MV* minute ventilation

^*^Statistically significant

### Interventricular delay

The median of the measured V-V sensing delay at implantation varied for the quadripolar LV lead depending on the used sensing electrode. When sensing occurred between RV and LV-E1 (distal LV electrode), the median was 75.7 ms (range 3–140 ms), and for sensing configuration RV–LV-E4 (most proximal LV electrode), the median was 95 ms (range 10–150 ms) (*n* = 34). Although the number of V-V delay data sets with reprogramming to MV ON at 1-month follow-up was small (*n* = 24), an increase in walking distance correlated with longer V-V timing. Within used cut-offs of 60 or 80 ms in V-V timing, a significant difference was seen between 1 and 3 months (60 ms, *p* = 0.034; 80 ms, *p* = 0.039).

### QRS duration

Data for ∆QRSD (*n* = 34) showed a shortening of the QRS duration after CRT implantation in 20/22 patients with left bundle branch block (LBBB) and in 6/12 patients with non-LBBB, signaling potentially beneficial CRT-P therapy. It could also be seen in patients with CI and with a wide (> 162 ms, median 182 ms) or narrower (≤ 162 ms, median 130 ms) QRS complex at baseline that both experienced improved mean walking distance between 1 and 3 months (wide 367.7 to 428.6 m, *p* = 0.011; narrower 359.0 to 433.6 m, *p* = 0.031) (Table 6).

**Table 6:** Comparison of changes in walking distance in patients with wider and narrower QRS width based on median split of measurements at enrollment

	QRS, *N* (median in ms)	Mean walking distance in m (1 month)	Mean walking distance in m (3 months)	*p*-value (1 to 3 months)	*p*-value (3 months)
All patients	21 (162)	363	431	*p* < 0.001	N/A
Patients with narrower QRS < 162 ms	11 (130)	359	434	*p* = 0.031	*p* = 0.60
Patients with wider QRS > 162 ms	10 (182)	368	429	*p* = 0.011	

## Discussion

CI defined as maximum HR < 100 bpm or 80% of APMHR after 6MWT was effective at identifying patients with a background of CI. Our main finding is that older HF patients with CI benefit from the use of a physiological activity MV sensor in CRT-P devices. The study showed beneficial clinical effects of using the MV sensor in CRT-P patients who could not reach a HR of 100 bpm or 80% of APMHR during 6MWT at 1-month follow-up. Better exercise tolerance was seen when the MV sensor was programmed ON in patients with CI between 1- and 3-month follow-ups. There was a substantial increase in the percentage of atrial pacing, maximum HR, and walking distance. Atrial pacing increased CI patients’ maximum HR in a similar way compared to non-CI patients with intrinsically driven atrial response. Our findings suggest that a systematic screening for CI may play a role in improving the clinical outcomes of older CRT-P patients and should be considered in clinical routine.

Increasing HR itself does not necessarily lead to higher exercise capacity. On the contrary, increasing HR with atrial pacing in patients with CI and HF with preserved ejection fraction (HFpEF) did not increase exercise tolerance [26]. This likely illustrates the difference between systolic and diastolic LV dysfunction. LV filling time is shortened by increased HR, which is essential in diastolic dysfunction. Furthermore, the PEGASUS trial did not demonstrate a difference in clinical outcomes between programming DDD-70, DDD-40, and DDDR-40 groups during CRT [27]. In that study, however, the amount of atrial pacing was almost identical in the DDD and DDDR groups indicating conservative programming of the sensor. In the CRT landmark studies, sensor-driven atrial pacing was not used [28–30]. In addition, in two of the studies, atrial pacing was avoided by either using VDD mode [28] or setting a lower rate of 40 bpm in DDD mode [30]. However, it is important to note that the patients in these early studies were much younger (median or mean 65–67 years) and thus less prone to CI than the patients in our study. There is concern that high amounts of atrial pacing predispose patients to AF. However, the recent randomized DANPACE II trial did not find any difference in the incidence of AF in sick sinus syndrome patients receiving either DDD-40 (atrial pacing 1%) or DDDR-60 (atrial pacing 49%) [31]. In addition, DDD-40 was associated with a higher incidence of syncope or presyncope. These results encourage the use of rate-response pacing when clinically indicated.

In the present study, cardiac output is impacted by the combined contributions of the sensor increasing HR and the effects of biventricular pacing through the action of the CRT-P system. Additional capacity for higher cardiac output is realized through synchronization of the ventricular contractions and increased HR driven by MV during physical activity. Taken together, this may explain why these patients achieve additional walking distance and why CI patients with both short and longer QRS durations benefit. The quickly gained capacity observed between 1 and 3 months suggests that there is probably a short-term cardiac output reserve that can be utilized by increased HR, which would support immediate rehabilitation possibilities and increased physical exercise shortly after CRT-P implantation. Increased capacity at 6 months post-implant and beyond is likely due to a different mechanism, possibly additional remodeling.

The non-CI subgroup received significantly less beta-blockers and ACE inhibitors prior to implantation of the device (Table 3). Medication may have had an impact on HR response and on the CI classification based on the criteria outlined in the protocol. Additionally, patients programmed to DDD did not show increased walking distance despite a numerical rise in HR. We would conclude that the reduced medication may help to avoid reduced HR response during exercise, but medication in combination with CRT and sensor support was associated with increased exercise capacity. Interestingly, a recent study involving patients with HFpEF and CI suggested that patients have improved functional capacity with the withdrawal of beta-blockers, especially in cases of low left ventricular end-systolic volume [32]. However, in HF with reduced LVEF, beta-blockers play a pivotal role as one of the cornerstones of treatment. Also, our current results underscore the importance of optimizing both medication and device therapy in managing HF effectively.

Previous work has shown a correlation between CRT response and prolonged interventricular delays (longer V-V timing). Patients with a V-V timing delay of ≥ 80 ms had significantly longer 6MWT distance improvements than patients with a V-V timing of < 80 ms, although patients with short V-V timing had longer walking distances overall. Longer V-V timings are usually associated with more advanced heart disease, thus a significant improvement for these sicker patients is encouraging. Notably, all the patients in the present study were elderly, meaning even small beneficial changes in their walking distances are clinically significant.

HRSc as a diagnostic device marker for CI was reduced (improved) in patients with DDDR-MV programming (*p* = 0.023). The largest impact was on 14 patients with poor HRSc (≥ 70%) (*p* ≥ 0.001). For patients with HRSc < 70% (less CI), the HRSc remained similar at 3 months (*p* = 0.68). 6MWT distance as exercise marker improved at 3 months for all 35 patients in the HRSc analysis (*p* = 0.006). Patients with HRSc < 70% demonstrated a statistically significant improvement (*p* = 0.031). However, patients with HRSc (≥ 70%) at 1-month follow-up showed less improvement, and the difference did not reach statistical significance at 3 months (*p* = 0.095). Poor HRSc may be associated with factors that limit exercise capacity, and reduction in HRSc may be associated with increases in activity at lower levels of exertion. In addition to 6MWT, HRSc is another useful tool for identifying CI, and it can be easily done using remote follow-up data.

The rising life expectancy is also increasing the need for research on therapy efficacy in elderly HF patients. More research on identifying and treating CI should be conducted in this growing population. Treatment of CI by MV sensor programming and optimization appears promising, but a randomized study would be necessary to document the potential impact on increased exercise capacity. Furthermore, particularly in this specific group of patients, a larger study evaluating the impact of treating CI on the quality of life would be valuable.

Conduction system pacing (CSP) is rapidly challenging biventricular pacing as the gold standard for resynchronization therapy [33–35]. A large, randomized study comparing CSP and biventricular pacing has recently started (NCT05650658). It remains to be seen whether CSP will offer a viable alternative to traditional CRT in older patients, who perhaps have more advanced disease in the conduction system and more fibrosis in the myocardium.

## Limitations

Our study does have noteworthy limitations. Firstly, due to the study design, the sample size included in the final analysis was relatively modest. Moreover, it is important to highlight that only the contribution of the MV sensor was evaluated in the study. Therefore, caution should be exercised when generalizing our findings to encompass the use of other types of sensors available in CRT devices. Despite these limitations, our study offers valuable data on the utilization of rate-response pacing as a part of CRT optimization among older patients who are frequently underrepresented in clinical studies.

## Conclusions

Older CRT-P patients under optimal medical therapy have an underestimated need for sensor-driven HR. Patients with MV-driven rate response and a high percentage of atrial pacing could increase their maximum HR in a similar way to patients with intrinsic-driven atrial response. The study showed beneficial clinical effects of using the MV sensor alone in CRT-P patients who could not reach 80% APMHR during 6MWT. In conclusion, our study demonstrates that old and very old CRT-P patients may benefit from the use of MV sensor.

## Supplementary Information

Below is the link to the electronic supplementary material.

Supplementary file1 (DOCX 30 KB)

## Data Availability

The data that support the findings of this study are available from Boston Scientific upon reasonable request. Limitation: The obtained patient consent doesn’t allow the secondary use of study data outside Rally CRT-P study protocol, including combination with other data sets.

## Abbreviations

6MWT: 6-Min walk test
AF: Atrial fibrillation
APMHR: Age-predicted maximal heart rate
CI: Chronotropic incompetence
CSP: Conduction system pacing
CRT: Cardiac resynchronization therapy
CRT-P: Cardiac resynchronization therapy pacemaker
ECG: Electrocardiogram
HF: Heart failure
HFpEF: Heart failure with preserved ejection fraction
HR: Heart rate
HRSc: Heart rate score
LBBB: Left bundle branch block
LV: Left ventricle
LVEF: Left ventricular ejection fraction
MV: Minute ventilation
NYHA: New York Heart Association
RV: Right ventricle
QRSD: QRS duration
∆QRSD: Shortening of the QRS duration
