# Supplementary material for: Minute ventilation sensor–driven rate response as a part of cardiac resynchronization therapy optimization in older patients
Source: J Interv Card Electrophysiol. 2024 Jun 24;67(9):2017–27. doi: 10.1007/s10840-024-01848-1 (PMC11711142; doi:10.1007/s10840-024-01848-1)
Supplement: Supplementary file 1 — Supplementary file1 (DOCX 30 KB) [file 10840_2024_1848_MOESM1_ESM.docx]

Supplementary **Table 1**. Overview of study follow-up and data collection.

| Procedure/  Assessment | Enrollment and consenting clinic visit  (required)  (≤ 30 days prior to implant procedure) | Implant procedure  (required)  (Day 0)  re-implant/  revision  (as needed) | Follow- Up Visits | | | | |
| --- | --- | --- | --- | --- | --- | --- | --- |
|  |  |  | Pre-discharge visit  (required)  (0-5 days post-implant procedure) | 1-month visit  (required)  (30 ± 15 days post-implant procedure) | 3-month visit  (required)  (91 ± 21 days post-implant procedure) | Unscheduled visit  (optional)  (according to center SOC or subject needs) | LATITUDE-based close-out*  (required)  (91-120 days post last enrollment) |
| Informed consent form, including informed consent signature and date | X | -- | -- |  | -- | -- | -- |
| Subject information, medical/device history | X | -- | -- |  | -- | -- | -- |
| Clinical assessment | X |  |  | O | O | -- | -- |
| Implant of CRT-P and implant measurements |  | X |  |  |  |  |  |
| 12 lead ECG recording | SOC |  |  |  | SOC |  |  |
| 12 lead ECG recording upload | X | -- |  |  | X | -- | -- |
| ECG 10s device based |  |  | X |  | X |  |  |
| LV pacing threshold for all available vectors |  |  | X |  | X | -- |  |
| Device assessment/  interrogation |  | X | X | X | X | -- |  |
| Device programming |  |  | X | X | X |  |  |
| 6-minute walk test |  |  |  | X | X |  |  |
| Device data collection  electronic upload |  |  | X | X | X |  |  |
| Current/  concomitant medications** | X |  | X | X | X |  |  |
| Adverse device events^\|\|^ and SAEs | -- | X | X | X | X | X | X |
| LATITUDE report on device data collection; battery status/diagnostic report |  |  | X | X | X | X | X |

Abbreviations: ECG: electrocardiogram; HF: heart failure; LV: Left ventricle; O: optional; POST: Post-Operative System Test; SAE: serious adverse event; SOC: standard of care; X: required; --: not required

*A 12-month post-implant data collection was required per protocol, and could be performed remotely without a site visit

**Only heart failure medication classes (Beta blockers, ACE inhibitors, diuretics, etc.); being administered or changes to administration of.

^||^To include collection of all USADEs, SADEs, ADEs, DDs; collection of all SAEs

**Supplementary Table 2.** Associated diseases and risk factors.

| **Risk Factor** | **N (% of 61 Enrolled Patients)** |
| --- | --- |
| Hypertension | 39 (63.9%) |
| Diabetes mellitus | 22 (36.1%) |
| Renal disease | 20 (32.8%) |
| Chronic pulmonary disease | 2 (3.3%) |
| Peripheral artery disease | 4 (6.6%) |
| Current cigarettes smoking | 3 (4.9%) |
| Other known malignancies (tumor, lymphoma, leukemia) | 3 (4.9%) |
| Any other chronic diseases | 14 (23.0%) |
| Other subject history | 31 (50.8%) |
| - Previous *stroke* - *Previous myocardial infarction* - Previous hospitalization for heart failure - Previous ablation (AV node, atrial fibrillation or atrial flutter) | *7* (11.5%)  16 (26.2%)  14 (23.0%)  6 (9.8%) |
| **Risk factor distribution in the patient cohort** |  |
| No risk factor | 2 (3.3%) |
| 1 risk factor | 9 (14.8%) |
| 2 risk factors | 8 (13.1%) |
| 3 risk factors | 17 (27.9%) |
| 4 risk factors | 14 (23.0%) |
| 5 risk factors | 6 (9.8%) |
| 6 risk factors | 4 (6.6%) |
| 7 risk factors | 1 (1.6%) |

The 9 risk factors at enrollment visit are: LVEF < 35%, NYHA class III or IV, (LBBB with QRS width > 130 ms) or any QRS > 150 ms, renal disease, diabetes mellitus, chronic atrial fibrillation, prior myocardial infarction, age > 70 years and current smoking.
